# Transwomen and the Metabolic Syndrome: Is Orchiectomy Protective?

**DOI:** 10.1089/trgh.2016.0016

**Published:** 2016-08-01

**Authors:** Michael D. Nelson, Lidia S. Szczepaniak, Janet Wei, Edward Szczepaniak, Francisco J. Sánchez, Eric Vilain, Jennifer H. Stern, Richard N. Bergman, C. Noel Bairey Merz, Deborah J. Clegg

**Affiliations:** ^1^ Barbra Streisand Women's Heart Center, Cedars-Sinai Heart Institute, Los Angeles, California.; ^2^ Biomedical Imaging Research Institute, Cedars-Sinai Medical Center, Los Angeles, California.; ^3^ MRS Consulting in Biomedical Research, Albuquerque, New Mexico.; ^4^ Department of Educational, School & Counseling Psychology, University of Missouri, Columbia, Missouri.; ^5^ Department of Human Genetics, Center for Gender-Based Biology, David Geffen School of Medicine at UCLA, Los Angeles, California.; ^6^ Department of Internal Medicine, Touchstone Diabetes Center, UT Southwestern Medical Center, Dallas, Texas.; ^7^ Diabetes and Obesity Research Institute, Cedars-Sinai Medical Center, Los Angeles, California.

**Keywords:** androgens, estrogens, insulin resistance, metabolic syndrome, orchiectomy, steatosis, transwomen

## Abstract

**Background:** Male-to-female transsexual women or *transwomen* who undergo cross-sex hormone treatments experience increased health-related risks (e.g., increased rates of cardiovascular disease and premature death). Yet, the exact mechanism by which altering biochemistry leads to metabolic impairment remains unclear. While much attention has been paid to cross-sex hormone therapy, little is known about the metabolic risk associated with orchiectomy.

**Methods:** To address the above limitation, we prospectively enrolled 12 transwomen: 4 who had undergone bi-lateral orchiectomy and 8 who had not. Both groups were using cross-sex hormones. Glucose tolerance was assessed using a standard 75g oral glucose tolerance test. Hepatic steatosis was assessed by ^1^H magnetic resonance spectroscopy. The amount of subcutaneous and visceral abdominal fat was determined from a single abdominal axial image at the level between the vertebral L2 and L3 bodies. Baseline venous fasting blood sampling was performed for measurement of hemoglobin A1c, glucose, insulin, sex hormones, and sex hormone binding globulin.

**Results:** The major novel findings were: (1) orchiectomy and cross-sex hormone therapy is associated with less hepatic steatosis and insulin resistance; (2) orchiectomy may be metabolically protective, and (3) circulating concentrations of sex hormones may be a major determinant of metabolic health in transwomen.

**Conclusions:** To our knowledge, this is the first study to suggest an independent and protective role of orchiectomy on the metabolic health of transwomen.

## Introduction

The United States has an estimated 8–16 million people (0.2–0.5% of the population) whose gender identity does not match their biological gender at birth.^1–5^ When the diagnostic criteria are met for gender dysphoria,^6^ individuals opt for interventions and procedures aimed at alleviating the incongruence between their gender identity and their biological sex; namely cross-sex hormones and sex-reassignment surgery.

The Endocrine Society cross-sex hormonal therapy guidelines suggest antiandrogen therapy combined with oral or transdermal estrogens to induce feminization for transwomen.^2^ Although these recommendations are established using best practice guidelines, little is known about relationships between hormonal and surgical therapy and cardiometabolic health. There are some observations suggesting that cross-sex hormone administration may be associated with the metabolic syndrome among transwomen^7^; however, the mechanistic underpinnings for these observations remain unclear and the role of orchiectomy unexplored. We hypothesized that cross-sex hormone therapy *without orchiectomy* would be associated with an adverse cardiometabolic status.

## Methods

The study was approved by the institutional review board at Cedars-Sinai Medical Center. All subjects provided written informed consent to participate.

Study participants (*n*=12) were recruited from a registry managed by the UCLA Center for Gender-Based Biology. Anthropometric and metabolic data before entry into the study were not annotated. The number of years following sex reassignment did not significantly differ among study participants. Eight of 11 participants were receiving intramuscular injections of estradiol valerate (10–40 mg, biweekly); whereas the other participants were receiving either transdermal estradiol (*n*=2) or oral estrogen compounds (*n*=1). Antiandrogen therapy (four of eight testes^+^ participants) consisted of spironolactone orally (100–200 mg/day). Four of the 12 participants had undergone bilateral orchiectomy (testes^−^) verified by magnetic resonance imaging and confirmed by a clinical radiologist. Exclusion criteria included the following: individuals with type 2 diabetes, age <18 years, body mass index (BMI) >44 kg/m^2^, contraindications to magnetic resonance imaging, and use of medications known to alter hepatic triglyceride content.

### Anthropometric measurements and OGTT

Height and weight were measured at the time of entry into the study. Fasting blood measurements for lipid profile, hemoglobin A1c, glucose, insulin, adiponectin, and sex hormones were performed. Insulin sensitivity was assessed following an oral glucose tolerance test (OGTT). Blood samples for glucose and insulin were obtained before and 10, 20, 30, 60, 90,120, and 180 min during the OGTT.

### Adiposity

Subcutaneous and visceral adiposity was determined from an axial abdominal image between the vertebral L2 and L3 bodies.^8^ Image analysis was performed by a blinded observer using Slice-O-Matic software (4.3 rev 10; Virtual Magic, Inc., Montreal, Canada). BMI was not known before enrollment in this study.

### ^1^H MRS of hepatic triglyceride content

**FIG. 1.**
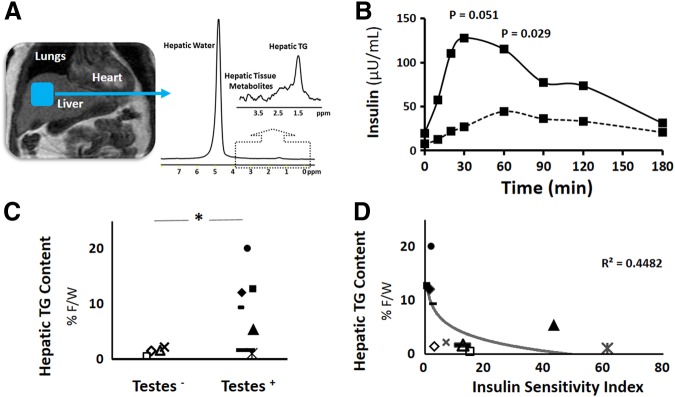
**(A)** High resolution image of the upper abdomen showing the region within the upper right hepatic lobe in which measurement of triglyceride (TG) content was obtained. Proton spectrum from the liver is also shown, illustrating the resonance peaks derived from hepatic water and TG. An expanded view of the spectrum is also included, highlighting the resonances from protons in the fatty acid chains. **(B)** Group average insulin response curves during oral glucose tolerance test. Solid line indicates subjects with testes (Testes^+^), whereas the dashed line indicates those subjects without testes (Testes^-^). **(C)** Individual hepatic TG content for subjects without testes (Testes^-^) and with testes (Testes^+^), expressed as ratio of fat to water (%). **(D)** Relationship between hepatic TG content and insulin sensitivity in all transwomen. *Denotes statistical significance, *p*<0.05.

Hepatic triglyceride content was quantified using ^1^H MRS at 3 Tesla. With the participant in the supine position, high-resolution, perpendicular images through the abdomen were collected to locate the liver. The volume for spectroscopic testing was selected at the upper right hepatic lobe using coronal, sagittal, and axial liver images with special attention not to include any visceral fat (Fig. 1A). Large volumes of interest (∼8 mL) were used to obtain average hepatic triglyceride levels. Spectroscopic data were collected as volunteers breathed freely, and the ^1^H MRS signal was triggered at end-expiration. Data were processed as previously described.^9–11^

### Biochemical analysis

#### Baseline and OGTT

Glucose was measured using a YSI 2300 or 2700 autoanalyzer (Yellow Springs Instruments, Yellow Springs, OH). Insulin was measured using an ELISA (EMD Millipore, Billerica, MA). Fasting glucose and insulin levels were used for homeostasis model assessment of insulin resistance,^12^ and the composite insulin sensitivity index was estimated from the glucose and insulin concentrations during the OGTT.^13^ The trapezoidal rule was used to calculate the areas under the curve (AUC) for glucose and insulin during the OGTT. Adiponectin was quantified by ELISA (Millipore Cat# EZHADP-61K).

#### Sex hormones

Hormone levels were quantified by immunoassay methods. Androstenedione, testosterone, estrone, and estradiol were measured in the same serum aliquot (0.5 mL) by radioimmunoassay following extraction with ethyl acetate:hexane (3:2) and Celite column partition chromatography using ethylene glycol as the stationary phase.^14–16^ Elution of the steroids off the column was carried out with isooctane, 40% toluene in isooctane, 15% ethyl acetate in isooctane, and 40% ethyl acetate in isooctane, respectively. DHEAS, cortisol, and SHBG were quantified on the Immulite 2000 analyzer (Siemens Healthcare Diagnostics, Deerfield, IL). The procedure for the DHEAS and cortisol assays is a solid-phase, competitive, chemiluminescent enzyme immunoassay. Sex hormone-binding globulin (SHBG), for the determination of free (nonprotein bound) estradiol and testosterone, was quantified by solid-phase, two-site chemiluminescent immunoassay on the Immulite analyzer. Free estradiol and testosterone were calculated with a validated algorithm based on derived equations.^17,18^ The algorithm uses measured concentrations of total estradiol or total testosterone and SHBG, an assumed average concentration of albumin (3.5 g/dL), and the appropriate affinity constants of SHBG and albumin for estradiol or testosterone.

### Statistical analysis

Data are reported as mean ± standard error unless otherwise stated. The prespecified primary analysis was comparison between transwomen with testes and those without using a *t*-test. The Mann–Whitney rank-sum test was used for nonnormally distributed data. Regression analysis assessed specific relationships. Secondary analysis revealed within group differences in circulating sex hormones, further dividing the subjects into three groups: (1) testes^−^, (2) testes^+^ with “low” testosterone concentrations, and (3) testes^+^ with “high” testosterone concentrations. To define differences between these three groups, a one-way analysis of variance was used. Rank sum tests were used if data were not normally distributed. To determine the overall influence of BMI and sex hormones on specific metabolic outcome variables, linear regression modeling was used, with log transformation applied to skewed distributions.

## Results

General group characteristics are presented in Table 1 for 12 transwomen—8 with testes (testes^+^) and 4 with orchiectomy (testes^−^). Participants did not differ by age, hemoglobin A_1c_, glucose, or glaciated hemoglobin. Testes^+^ transwomen trended toward having a higher BMI (*p*<0.1), but had significantly higher total, and subcutaneous adiposity when compared to testes^−^ transwomen (*p*<0.05) (Table 1).

**Table 1.** **Baseline Characteristics**

	Transsexual women testes^+^	Transsexual women testes^−^
*n*	8	4
Ethnicity, C/H/AA	2/2/0	2/6/0
Age, years	39±10	40±5
Height, cm	176.0±7.6	174.8±6.0
Weight, kg	104.7±31.9	76.6±8.0
BMI, kg/m^2^	33.3±7.7	25.1±2.8^a^
Hepatic triglyceride, fat/H_2_O	1.5±0.7	8.9±6.8
Visceral fat, cm^3^	193.5±117.0	79.9±26.6
Subcutaneous fat, cm^3^	319.6±120.7	129.9±29.2^b^
Whole body area, cm^3^	894.3±349.0	560.5±69.8^a^
Total fat area, cm^3^	513.1±226.0	209.7±52.7^b^
Visceral fat, %	20±7	14±3
Subcutaneous fat, %	36±5	23±3^b^
Total fat, %	56±4	37±5^b^
Total glycated Hgb	5.9±0.7	5.3±0.6^a^
Hgb A_1c_	5.2±0.4	4.8±0.3
Fasting glucose, mg/dL	91.5±7.5	87.9±4.1
Fasting insulin, μU/mL	19.4±15.6	7.7±7.7^a^
HOMA-IR	4.6±3.7	1.7±1.8^a^
SHBG, nmol/L	64.3±42.6	126.5±18.0^b^
Testosterone, ng/dL	71.9±71.5	16.3±4.8
Free testosterone, pg/mL	15.2±15.4	1.6±0.5^b^
FT + AT, ng/mL	37.4±37.7	4.0±1.1^b^
Cortisol, μg/dL	16.5±5.4	14.4±3.9
DHEAS, μg/dL	106.9±60.3	85.7±34.4
Androstenedione, pg/mL	521.3±202.2	531.7±204.0
Estrone, pg/mL	204.2±181.9	256.2±127.9
Estradiol, pg/mL	209.3±235.0	500.7±197.3
Free estradiol, pg/mL	4.6±5.8	7.5±2.8
FE2 + AE2, pg/mL	117.6±146.7	191.1±70.6
Adiponectin, μg/mL	11.5±6.7	5.9±4.2^a^

Data reported as mean±SD.

*p-*Value indicates differences between groups with and without testes.

^a^ *p*≤0.10.

^b^ *p*<0.05.

AE2, albumin-bound estradiol; AT, albumin-bound testosterone; BMI, body mass index; C/H/AA, Caucasian/Hispanic/African American; FE2, free estradiol; FT, free testosterone; Hgb, hemoglobin; HOMA-IR, homeostasis model assessment of insulin resistance; SHBG, sex hormone-binding globulin.

Testes^+^ transwomen tended to have an overall heightened insulin response to the glucose challenge (Fig. 1B), when compared to testes^−^ transwomen. Despite similar plasma glucose levels during the OGTT (17823±859 vs. 20380±1263 mg/dL/180 min, *p*<0.21), the AUC for insulin was more than twofold higher in testes^+^ transwomen compared to testes^−^ transwomen (14235±4694 vs. 5491±1538 μU/mL/180 min; *p*=0.283), and plasma insulin was significantly elevated in the testes^+^ transwomen compared to testes^−^ transwomen at 30 and 60 min (Fig. 1B). In line with this insulin resistance, plasma adiponectin was also nearly twofold lower in testes^+^ transwomen versus testes^−^ transwomen (5.9±4.2 vs. 11.5±6.7 μg/mL; *p*=0.101).

Hepatic triglyceride content was significantly elevated in all, but two of the testes^+^ transwomen compared to testes^−^ transwomen (Fig. 1C). Consistently, those individuals with the greatest levels of hepatic triglyceride also were the most insulin resistant (Fig. 1D, *p*<0.028).

As expected, serum sex-hormone levels differed between testes^+^ and testes^−^ transwomen. Testes^+^ transwomen had higher levels of testosterone, free testosterone and bioavailable testosterone (free testosterone plus albumin-bound testosterone), and lower levels of sex hormone-binding globulin, compared to testes^−^ transwomen (Table 1). In addition, estradiol was lower in the testes^+^ transwomen compared to testes^−^ transwomen (Table 1). The groups did not differ in cortisol, DHEAS, androstenedione, or estrone levels (Table 1).

**FIG. 2.**
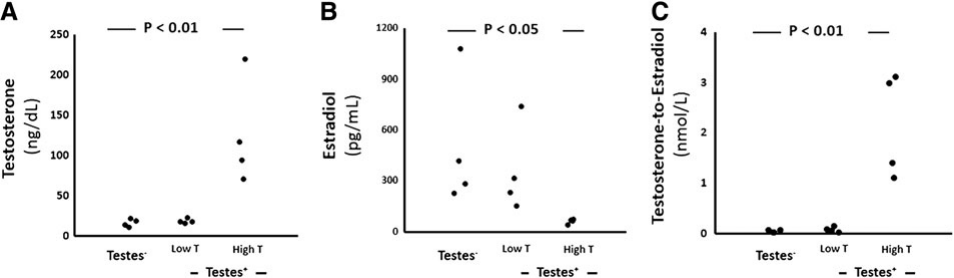
Within-group differences in sex hormone levels **(A–C)** divided by testosterone levels.

In addition, we found significant within-group differences, independent of hormonal therapy. In particular, four of the eight testes^+^ transwomen had significantly higher levels of testosterone when compared to the remaining testes^+^ or testes^−^ transwomen (Fig. 2A–C, High T group). Moreover, the four transwomen with the highest testosterone levels (High T group) also had the lowest levels of estradiol (Fig. 2B) and SHBG (126.5±9.0, 95.1±19.2, 33.4±7.3 nmol/L; testes^−^, testes^+^ low T, testes^+^ high T, respectively). As a result, the testosterone-to-estradiol ratio was significantly elevated in the High T group (by 53 times), when compared to the other two groups (Fig. 2C).

**FIG. 3.**
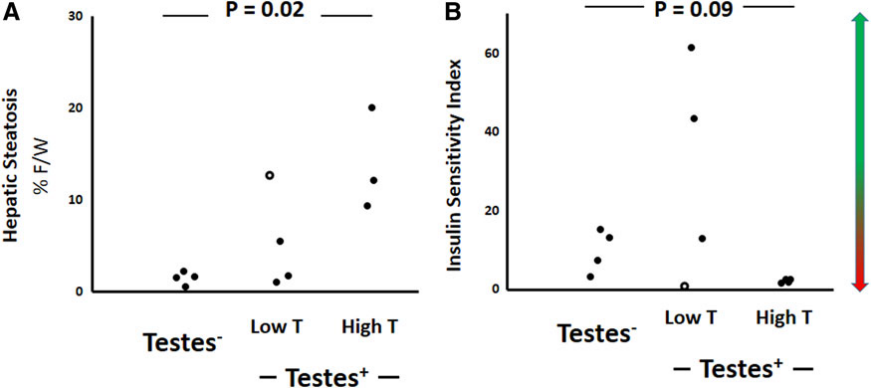
Within-group differences in hepatic steatosis **(A)** and insulin sensitivity **(B)** when divided by testosterone levels. The arrow next to Panel B (Insulin Sensitivity Index) provides directionality, with green pointing in the direction of “good” insulin sensitivity and red signifying “insulin resistance.”

Importantly, transwomen with the highest levels of testosterone also tended to have the highest hepatic steatosis (Fig. 3A) and insulin resistance (Fig. 3B). One participant (open circle, Fig. 3) with the highest hepatic steatosis in the “Testes^+^, Low T” group also had the greatest insulin resistance within that group, further strengthening the potential mechanistic relationship between these two outcomes in this cohort.

To further differentiate the influence of body adiposity and circulating testosterone on hepatic steatosis and insulin resistance, we performed linear regression modeling. A regression of insulin sensitivity on both testosterone and BMI showed a significant model fit, but neither explanatory factor was found to be individually significant (*R*^2^=0.4989, *p*=0.045). In contrast, a regression of hepatic steatosis on both testosterone and BMI showed a significant model fit, with only testosterone being individually significant (*R*^2^=0.67, *p*=0.012).

## Discussion

To our knowledge, this is the first study to specifically compare transwomen with and without orchiectomy related to cardiometabolic measures, including hepatic steatosis, insulin sensitivity, and serum hormonal levels. We report three major new findings: (1) orchiectomy and cross-sex therapy is associated with less hepatic steatosis and insulin resistance when compared to cross-sex hormone therapy alone, (2) insulin resistance is positively related to hepatic steatosis—a finding not previously reported in this population, and (3) elevations in serum-*free* testosterone levels are positively associated with hepatic steatosis and insulin resistance in transwomen. Taken together, these data suggest a balance between *free* testosterone levels, and estradiol may represent an important biomarker in transwomen for cardiometabolic risk.

The relationship between serum sex hormones and cardiometabolic health is supported by the subset of testes^+^ transwomen who had elevated free testosterone levels compared to the other participants. These participants also had the highest levels of hepatic steatosis, the greatest measured insulin resistance, and the lowest levels of estrogens and SHBG. Further work is needed to determine if these factors are mechanistically related or causally associated. Importantly, we observed that orchiectomy was associated with less liver steatosis and insulin sensitivity, despite cross-sex hormone treatment. Admittedly, the duration, dosage, and timing of cross hormone therapy did differ among our subjects; however, our sample size is small enough that we were unable to power the study adequately to factor in these potential confounding variables. Circulating levels of adiponectin, an insulin sensitizing, antiapoptotic, and anti-inflammatory adipokine, is typically inversely correlated with adiposity and is higher in women than men.^19^ While the mechanism for this sex difference is not entirely elucidated, circulating testosterone has been shown to inhibit the secretion of adiponectin from the adipocyte.^20,21^

The ratio of testosterone and estradiol has been proposed as a marker of cardiometabolic health.^22–24^ In a study by Gong et al.^22^ a high testosterone-to-estradiol ratio was associated with cerebrovascular disease. Likewise, Zheng et al. found the testosterone-to-estradiol ratio to be nearly twice higher in men with coronary heart disease, compared to age-matched controls.^24^ In our study, we found that transsexual women with an elevated testosterone had the lowest levels of estradiol, resulting in a testosterone-to-estradiol ratio 53 times higher than either of the other two groups, suggesting that the balance between testosterone and estradiol may be more important than absolute levels of either hormone for cardiometabolic health. Importantly, the duration and type of estrogen supplementation did not differ across the groups; however, utilization of estrogens was self-reported.

This clinical case series is not without limitation. First, our small sample size precludes definitive conclusions and must be interpreted with caution. However, we observed group differences, despite the small sample size, suggesting that the associations—if real—are very robust. Our study is cross-sectional, thus precluding us from making causative conclusions regarding the relationship and the mechanisms involved. In addition, information such as metabolic health and BMI before gender reassignment and enrollment in our study is largely unknown and admittedly may confound interpretations of our findings. We therefore believe that our small observational trial provides novel insight that requires larger longitudinal investigations to confirm our initial observations and to specifically evaluate the prognostic importance of these findings on the cardiometabolic health and cardiovascular disease events in transwomen. BMI was higher in the testes^+^ transwomen; however, because the transwomen with the highest testosterone levels also had the highest BMI and subcutaneous and total fat percentage, we find these data important because normally high BMI is associated with low testosterone, yet in the group with the highest testosterone, we observed the highest BMI. Admittedly, we do not know the BMI of the individuals at the time of gender reassignment if there is any impact of this on our results.

## Perspectives and Conclusions

The Guidelines on Endocrine Treatment of Transsexuals concludes by recommending the need for rigorous evaluations of the safety, effectiveness, and long-term impact of endocrine protocols in transgenders.^25^ In this study, we show that (1) orchiectomy may be metabolically protective and (2) the circulating ratio of sex hormones may be a major determinant of metabolic health in transwomen. Future research is indeed warranted. Importantly, additional longitudinal and prospective studies should be conducted in this understudied population. Implications from our findings may suggest in those individuals who elect to maintain their testes; additional pharmacology may be required to suppress endogenous T levels, such as GnRH antagonists, to maintain a more favorable T/E ratio and enhance cardiometabolic health.

## Abbreviations Used

AUC: areas under the curve
BMI: body mass index
OGTT: oral glucose tolerance test
SHBG: sex hormone-binding globulin

## References

[1] California Department of Finance. Race/Ethnic Population with Age and Sex Detail, 2000–2050. Sacramento, CA, 2007

[2] Conron K, Scott G, Stowell G, Landers S Transgender health in Massachusetts: results from a household probability sample of adults. Am J Public Health. 2012;102:118–122 22095354 10.2105/AJPH.2011.300315 PMC3490554

[3] Gates G How Many People Are Lesbian, Gay, Bisexual, and Transgender? Los Angeles, CA: UCLA School of Law, The Williams Institute, 2011

[4] Reed B, Rhodes S, Schofield P, Wylie K Gender Variance in the UK: Prevelance, Incidence, Growth, and Geographic Distribution. United Kingdom: Gender Identidy Research and Education Society, 2009

[5] Horton MA The Prevalence of SRS Among US Residents. Austin, Texas: Out & Equal Workplace Summit, 2008

[6] American Psychiatric Association: Diagnostic and Statistical Manual of Mental Disorders, 5th ed. Arlington, VA, 2013

[7] Gooren LJ, Giltay EJ, Bunck MC Long-term treatment of transsexuals with cross-sex hormones: extensive personal experience. J Clin Endocrinol Metab. 2008;93:19–25 17986639 10.1210/jc.2007-1809

[8] Abate N, Garg A, Coleman R, et al. Prediction of total subcutaneous abdominal, intraperitoneal, and retroperitoneal adipose tissue masses in men by a single axial magnetic resonance imaging slice. Am J Clin Nutr. 1997;65:403–408 9022523 10.1093/ajcn/65.2.403

[9] Szczepaniak LS, Nurenberg P, Leonard D, et al. Magnetic resonance spectroscopy to measure hepatic triglyceride content: prevalence of hepatic steatosis in the general population. Am J Physiol. 2005;288:E462–E468 10.1152/ajpendo.00064.2004 15339742

[10] Szczepaniak LS, Babcock EE, Schick F, et al. Measurement of intracellular triglyceride stores by H spectroscopy: validation in vivo. Am J Physiol. 1999;276:E977–E989 10329993 10.1152/ajpendo.1999.276.5.E977

[11] Szczepaniak LS, Victor RG, Mathur R, et al. Pancreatic steatosis and its relationship to beta-cell dysfunction in humans. Diabetes Care. 2012;7:1–7 10.2337/dc12-0701 PMC3476895 22968187

[12] Mathews D, Hosker J, Rudenski A, et al. Homeostasis model assessment: insulin resistance and beta-cell function from fasting plasma glucose and insulin concentrations in man. Diabetologia. 1985;28:412–419 3899825 10.1007/BF00280883

[13] Matsuda M, DeFronzo RA Insulin sensitivity indices obtained from oral glucose tolerance testing: comparison with the euglycemic insulin clamp. Diabetes Care. 1999;22:1462–1470 10480510 10.2337/diacare.22.9.1462

[14] Goebelsmann U, Horton R, Mestman J, et al. Male pseudohermaphroditism due to testicular 17-hydroxysteroid dehydrogenase deficiency. J Clin Endocrinol Metab. 1973;36:86 7 10.1210/jcem-36-5-867 4349047

[15] Goebelsmann U, Arce J, Thorneycroft I, Mishell DJ Serum testosterone concentrations in women throughout the menstrual cycle and following hCG administration. Am J Obstet Gynecol. 1974;119:44 5 10.1016/0002-9378(74)90199-9 4842588

[16] Probst-Hensch NM, Ingles SA, Diep AT, et al. Aromatase and breast cancer susceptibility. Endocr Relat Cancer. 1999;6:165–173 10731105 10.1677/erc.0.0060165

[17] Rinaldi S, Geay A, Déchaud H, et al. Validity of free testosterone and free estradiol determinations in serum samples from postmenopausal women by theoretical calculations. Cancer Epidemiol Biomarkers Prev. 2002;11:1065–1071 12376508

[18] Vermeulen A, Verdonck L, Kaufman JM A critical evaluation of simple methods for the estimation of free testosterone in serum. J Clin Endocrinol Metab. 1999;84:3666–3672 10523012 10.1210/jcem.84.10.6079

[19] Bottner A, Kratzsch J, Muller G, et al. Gender differences of adiponectin levels develop during the progression of puberty and are related to serum androgen levels. J Clin Endocrinol Metab. 2004;89:4053–4061 15292348 10.1210/jc.2004-0303

[20] Nishizawa H, Shimomura I, Kishida K, et al. Androgens decrease plasma adiponectin, an insulin-sensitizing adipocyte-derived protein. Diabetes. 2002;51:2734–2741 12196466 10.2337/diabetes.51.9.2734

[21] Xu A, Chan KW, Hoo RLC, et al. Testosterone selectively reduces the high molecular weight form of adiponectin by inhibiting its secretion from adipocytes. J Biol Chem. 2005;280:18073–18080 15760892 10.1074/jbc.M414231200

[22] Gong Y, Xiao H, Li C, et al. Elevated T/E2 ratio is associated with an increased risk of cerebrovascular disease in elderly men. PLoS One. 2013;8:e6159 8 10.1371/journal.pone.0061598 PMC3634802 23637864

[23] Myking OLE, Aakvaag ASBJ, Digranes O Androgen−oestrogen imbalance in men with chronic alcoholism and fatty liver. Alcohol Alcohol. 1987;22:7–15 3593486

[24] Zheng H, Li Y, Dai W, et al. Imbalance of testosterone/estradiol promotes male CHD development. Biomed Mater Eng. 2012;22:179–185 22766718 10.3233/BME-2012-0705

[25] Hembree WC, Cohen-Kettenis P, Delemarre-van de Waal H, et al. Endocrine treatment of transsexual persons: an endocrine society clinical practice guideline. J Clin Endocrinol Metab. 2009;94:3132–3154 19509099 10.1210/jc.2009-0345

